# Depression, anxiety, and health-related quality of life in normal weight, overweight and obese individuals with diabetes: a representative study in Germany

**DOI:** 10.1007/s00592-024-02248-7

**Published:** 2024-03-02

**Authors:** Benedict Herhaus, Johannes Kruse, Andreas Hinz, Elmar Brähler, Katja Petrowski

**Affiliations:** 1 Medical Psychology and Medical Sociology, University Medical Center Mainz, Johannes Gutenberg University of Mainz, Duesbergweg 6, 55128 Mainz, Germany; 2 Department of Psychosomatic Medicine and Psychotherapy, Philipps University Marburg, Marburg, Germany; 3 Department of Psychosomatic Medicine and Psychotherapy, Justus Liebig University Giessen, Giessen, Germany; 4 Department of Medical Psychology and Medical Sociology, University of Leipzig, Leipzig, Germany; 5 Integrated Research and Treatment Center Adiposity Diseases, Behavioral Medicine Unit, Department of Psychosomatic Medicine and Psychotherapy, Leipzig University Medical Center, Leipzig, Germany; 6 Department of Psychosomatic Medicine and Psychotherapy, University Medical Center Mainz, Johannes Gutenberg University Mainz, Mainz, Germany

**Keywords:** Diabetes, Obesity, Depression, Anxiety, Health-related quality of life

## Abstract

**Objective:**

Diabetes in the course of lifetime is related to a higher risk for mental disorders. The present study addresses the comparison of individuals with diabetes and non-diabetic individuals in depressive symptoms, generalized anxiety symptoms, and health-related quality of life. Furthermore, mediator effect of BMI and health-related quality of life (HRQOL) on the association between diabetes, depression, and generalized anxiety was analyzed.

**Methods:**

In this cross-sectional study, the three questionnaires PHQ-9, GAD-7, EQ-5D-5L were measured in a representative sample of the German population (N = 2386). In addition, the presence of diabetes and BMI were assessed via self-report.

**Results:**

There were higher values in depressive and anxiety symptoms as well as lower score in HRQOL in individuals with diabetes compared to non-diabetic individuals. Obese individuals with diabetes showed the highest rates in depressive symptoms and generalized anxiety as well as lowest score in HRQOL. With regard to the mediator analyses, association between diabetes, depressive symptoms, and anxiety symptoms is partially mediated by the BMI and fully mediated by the HRQOL.

**Conclusions:**

In conclusion, individuals with diabetes have an increased risk in the development of depressive and anxiety symptoms as well as lower health-related quality of life. Future research and strategies in the public health policies among individuals with diabetes should take into account that the association between diabetes, depression, and anxiety is mediated by BMI and HRQOL.

**Supplementary Information:**

The online version contains supplementary material available at 10.1007/s00592-024-02248-7.

## Introduction

Approximately 7% of adults aged 18 years and above suffer from type 1 and type 2 diabetes mellitus in Germany [1, 2] and the global diabetes prevalence will rise to 700 millions by 2045 [3]. Diabetes over the course of one's lifetime is related to nephropathy, cardiovascular disease (CVD), and higher mortality [4, 5]. Besides somatic disease, having diabetes is also associated to the development of mental disorders.

There is evidence that diabetes doubled the likelihood of having depression [6]. A cross-sectional study in Ireland showed higher depression symptoms in 2049 individuals with type 1 and type 2 diabetes [7]. With regard to the other direction, a meta-analysis from Rotella and Mannucci [8] revealed increased risk of developing diabetes in individuals suffering from depression. Furthermore, a systemic review demonstrated a bidirectional association between diabetes and depression [9]. Therefore, these findings showed that depression is a predicting factor of developing diabetes and the presence of diabetes increases the risk of depression.

With regard to generalized anxiety, meta-analyses of cross-sectional studies showed that individuals with diabetes have an increased risk of developing an anxiety disorder or elevated anxiety symptoms [10, 11]. In contrast, a meta-analysis of prospective studies demonstrated that anxiety over the course of one's lifetime is related to diabetes, but not the other way [12]. Therefore, these findings indicated that anxiety is a predictive factor for the onset of diabetes, and having diabetes elevates the likelihood of developing anxiety symptoms. But no study found a bidirectional association between diabetes and anxiety.

To prevent depression and anxiety in diabetes, it is necessary to clarify underlying factors of the concerned association. The self-perception of the health-related quality of life (HRQOL) and changes in BMI over the course of one's lifetime are both related to depressive and anxiety symptoms [13–15]. Both factors have also an impact on developing mental disorders [16, 17]. The HRQOL is reduced in individuals with diabetes compared to non-diabetic individuals [18] and poor self-rated health is a predicting factor of developing depressive and anxiety symptoms in individuals with diabetes [16, 19, 20]. Furthermore, obesity as the leading risk factor for type 2 diabetes [21] affects the HRQOL, and the HRQOL also mediates the association between BMI, depressive symptoms and anxiety symptoms [14].

In conclusion, in the course of lifetime diabetes is related to a higher risk for depressive and anxiety symptoms. Most studies investigated the differences in mental symptoms in individuals with diabetes and non-diabetic individuals, but often missed to explore the mechanism through mediating variables. Understanding the mediating role of HRQOL and obesity in the association of diabetes and depressive/anxiety might improve strategies in the public health policies among individuals with diabetes.

Therefore, in the current cross-sectional study, depressive symptoms, generalized anxiety, HRQOL, and self-reported BMI were collected in individuals with diabetes and non-diabetic individuals in a representative sample of the German population. Based on the current state of research [7, 10, 11, 22], we hypothesized higher values in depressive symptoms and generalized anxiety in individuals with diabetes compared to non-diabetic individuals (hypothesis 1). Regarding the link between diabetes and HRQOL [18], we hypothesized that individuals with diabetes exhibit lower values in HRQOL compared to non-diabetic individuals (hypothesis 2). Regarding the link between diabetes, symptoms of depression/anxiety, HRQOL, and BMI [15, 16, 19, 20], we hypothesized that the BMI and HRQOL mediate the association between diabetes, depressive symptoms and generalized anxiety symptoms (hypothesis 3).

## Methods

### Design and participants

This cross-sectional survey of a representative sample of the German population was carried out by the demographic research company USUMA (Berlin, Germany). The households and participants were selected by random-route sampling in line with the ADM [23]. Thereby, Germany was divided into 258 sampling areas, encompassing both Eastern and Western regions, as well as various rural and urban zones across the country. The random selection of household members was then conducted within these designated areas and face-to-face interviews were conducted by trained interviewers. All participants (N = 2386) were aged 18 years and above with a mean age of 50.70 ± 17.34 years, and 52% of the represented were female (48% male). The study was performed in accordance with the declaration in agreement with the Helsinki Declaration and was approved by the Ethical Committee of the Medical Faculty, University of Leipzig, Germany (072-11-07032011). All participants gave written informed consent prior to their participation.

### Questionnaires

#### Psychological assessment PHQ-9

Depression severity during the past two weeks was measured by the Patient Health Questionnaire (PHQ-9) of Löwe et al. [24] The questionnaire consists of nine items with a four-point rating scale (0 ‘not at all’ to 3 ‘nearly every day’). The sum ranges between 0 and 27 where a higher overall score indicates higher depressive symptoms. In the current sample, the internal consistency exhibited good reliability values (Cronbach’s α of 0.87) for the PHQ-9.

#### GAD-7

The GAD-7 of Spitzer et al. [25] assess the generalized anxiety and its severity during the past two weeks. The questionnaire is based on seven items with a four-point Likert scale (range 0–3) and results in a sum value between 0 and 21 (higher overall score indicates higher anxiety symptoms). In the current sample, the internal consistency exhibited good reliability values of Cronbach’s α of 0.87 for the GAD-7.

#### EQ-5D-5L

Current health-related quality of life (HRQOL) was measured by the EuroQol-5Dimension-5Level (EQ-5D-5L) of Herdman et al. [26]. The five items represent the five dimensions mobility, self-care, usual activities, pain/discomfort, and anxiety/depression. Calculating of the sum score was carried out using the formula by Hinz et al. [26]. A higher sum score indicates higher HRQOL. In diverse studies and in different subgroups reliability and validation of the EQ-5D-5L were proven [28, 29]. In the current sample, the internal consistency exhibited good reliability values of Cronbach’s α of 0.82 for the EQ-5D-5L.

#### Demographic questionnaire and body mass index

The presence of diabetes without distinction of type 1 and type 2 as well as current body weight and height were asses via self-report. Based on the calculated BMI (kg/m^2^), the participants were divided into the three BMI classes: normal weight (18.5 ≥ BMI < 25), overweight (25 ≥ BMI < 30), and obesity (BMI ≥ 30). Further information about age, gender, marital status, work status and household income were collected by a standardized questionnaire used in previous surveys [30].

### Statistical analysis

All statistical analyses were conducted using SPSS Statistics version 27 (IBM, Chicago, IL, USA). The differences between the individuals with diabetes and without diabetes in demographic characteristics were tested by Chi-square test and ANOVA. ANOVA´s were applied to test differences in depressive symptoms, generalized anxiety symptoms and HRQOL between the group of individuals with diabetes and without diabetes as well as between the three BMI classes normal weight, overweight, and obesity. The assumption of sphericity was controlled by Mauchly’s test. Whenever necessary, the ANOVA results were corrected by Greenhouse–Geisser. In addition, post hoc tests (Bonferroni-Holm corrections) were performed to assess differences between individual BMI classes.

To analyze whether BMI and/or HRQOL mediated the association between diabetes, depressive symptoms and generalized anxiety symptoms, mediation analyses were performed. Age and gender were included as covariates to control potential influencing socio-demographic factors. Visual inspection of the scatterplots after LOESS smoothing was carried out to test the linearity of all included variables. For the mediation analyses, PROCESS macro by Hayes [30] was performed, which uses ordinary least squares regression, yielding unstandardized path coefficients for total, direct, and indirect effects. Bootstrapping with 5000 samples together with heteroscedasticity consistent standard errors were applied to calculate the confidence intervals and inferential statistics [32]. Significance was assumed if the confidence interval of the indirect effect did not include zero.

## Results

### Sample characteristics

A description of the sociodemographic data of the study population is given in Table 1. There were significant differences between individuals with diabetes and non-diabetic individuals in the variables BMI class (*χ*^2^ = 76.1, *df* = 2, *p* ≤ 0.001, Cramér’s *V* = 0.179), age (*F*_(1, 2384)_ = 195.8, *p* ≤ 0.001, *η*^2^ = 0.076), age groups (*χ*^2^ = 217.1, *df* = 5, *p* ≤ 0.001, Cramér’s *V* = 0.302), marital status (*χ*^2^ = 96.9, *df* = 3, *p* ≤ 0.001, Cramér’s *V* = 0.202), work status (*χ*^2^ = 214.4, *df* = 4, *p* ≤ 0.001, Cramér’s *V* = 0.300) and household income (*χ*^2^ = 28.4, *df* = 3, *p* ≤ 0.001, Cramér’s *V* = 0.109). No difference was observed between the groups regarding sex (*χ*^2^ = 1.1, *df* = 1, *p* = 0.31).

**Table 1:** Sociodemographic description of the study population

BMI-classification	Diabetes *N* = 206		Non-diabetes *N* = 2180		Statistical testing
	N	(%)	N	(%)	
BMI classes					*x*^2^ = 76.060, *p* ≤ 0.001, Cramér ‘s V = 0.179
Normal weight	53	(26%)	1150	(53%)	
Overweight	111	(54%)	875	(40%)	
Obesity	42	(20%)	155	(7%)	
Sex					*x*^2^ = 1.120, *p* = 0.31
Female	115	(56%)	1133	(52%)	
Male	91	(44%)	1047	(48%)	
Age, M ± SD	66.23	± 11.87	49.23	± 17.05	*F*_(1,2384)_ = 195.759, *p* ≤ 0.001, *η2* = 0.076
Age groups					*x*^2^ = 217.097, *p* ≤ 0.001, Cramér’s V = 0.302
18–30 years	2	(1%)	370	(17%)	
31–40 years	6	(3%)	330	(15%)	
41–50 years	17	(8%)	464	(21%)	
51–60 years	26	(13%)	405	(19%)	
61–70 years	64	(31%)	329	(15%)	
> 71 years	91	(44%)	282	(13%)	
Marital status					*x*^2^ = 96.903, *p* ≤ 0.001, Cramér’s V = 0.202
Single	15	(7%)	542	(25%)	
Married	108	(53%)	1130	(52%)	
Divorced	17	(8%)	276	(13%)	
Widowed	66	(32%)	232	(10%)	
Work status					*x*^2^ = 214.424, *p* ≤ 0.001, Cramér ‘s V = 0.300
Employed	26	(13%)	1196	(55%)	
Unemployed	17	(8%)	152	(7%)	
Pensioners	155	(75%)	600	(28%)	
Not employeed	8	(4%)	96	(4%)	
In education	0	(0%)	136	(6%)	
Household income					*x*^2^ = 28.358, *p* ≤ 0.001, Cramér’s V = 0.109
< 1250 €/month	76	(37%)	484	(22%)	
1250–2500 €/month	91	(44%)	999	(46%)	
> 2500 €/month	34	(17%)	638	(29%)	
No Information	5	(2%)	59	(3%)	

### Depressive symptoms—PHQ-9

**Fig. 1:**
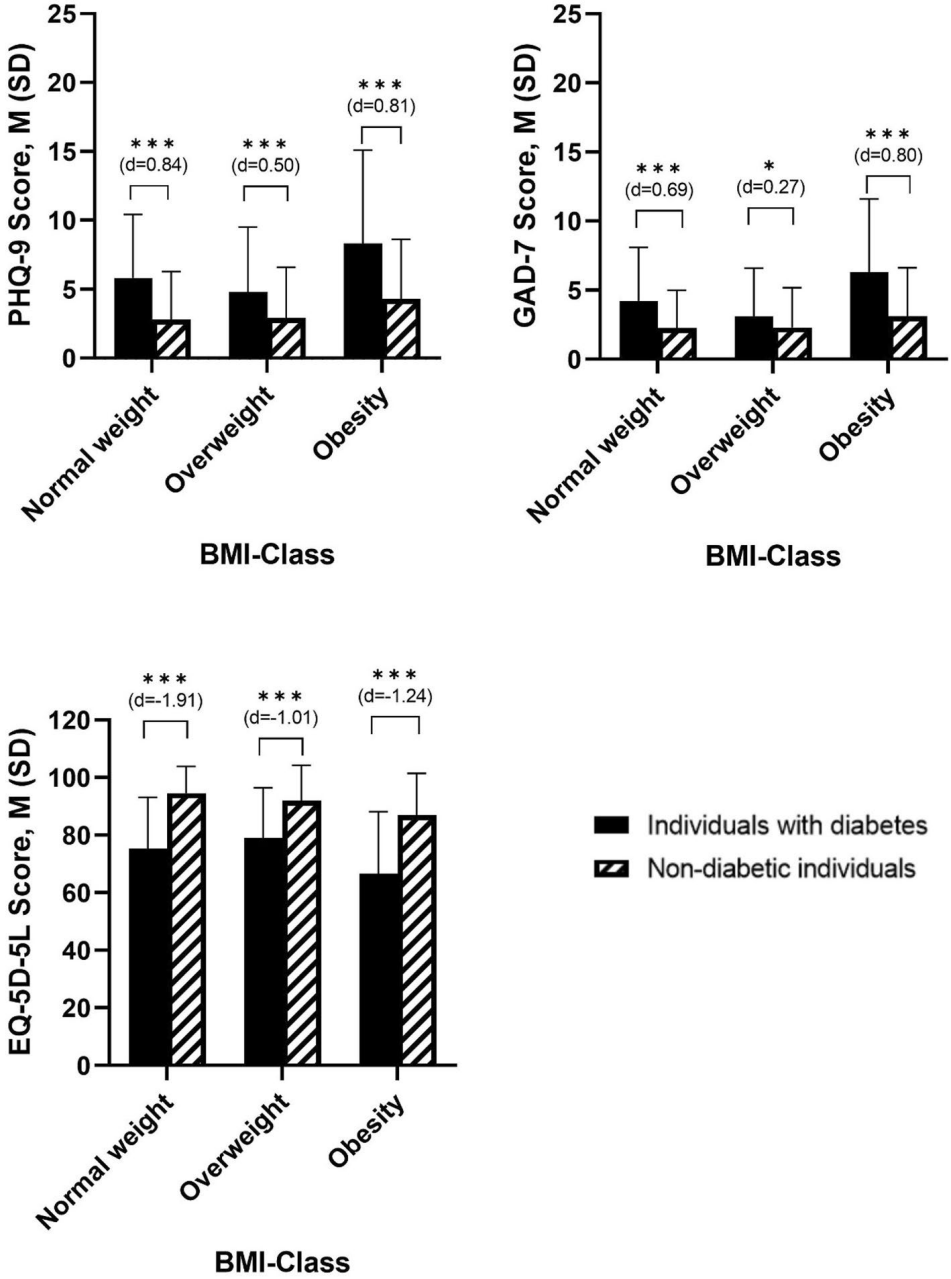
Comparison of individuals with diabetes and non-diabetic individuals across different BMI classes in in the PHQ-9, GAD-7 and EQ-5D. *: *p* ≤ 0.05; ***: *p* ≤ 0.001

The results in Table 2 demonstrated significant differences in the PHQ-9 score in the factor diabetes (*F*_(1, 2380)_ = 90.9, *p* ≤ 0.001, *η*^2^ = 0.037) and in the factor BMI class (*F*_(2, 2380)_ = 20.5, *p* ≤ 0.001, *η*^2^ = 0.017) with significant interaction effect diabetes x BMI class (*F*_(1, 2380)_ = 4.0, *p* ≤ 0.05, *η*^2^ = 0.003). Obese individuals have the strongest increase in the PHQ-9 score from non-diabetes to diabetes (see Supplemental Fig. 1). As shown in Fig. 1, further post-hoc tests showed that in all three BMI classes individuals with diabetes revealed significant higher PHQ-9 scores compared to non-diabetic individuals (normal weight: *p* ≤ 0.001; overweight: *p* ≤ 0.001, obesity *p* ≤ 0.001).

**Table 2:** Differences of individuals with diabetes and non-diabetic individuals as well as BMI class differences in the PHQ-9, GAD-7 and EQ-5D-5L

Variable	PHQ-9	GAD-7	EQ-5D-5L
Diabetes	*F*_(1,2380)_ = 90.867, *p* ≤ 0.001	*F*_(1,2380)_ = 64.887, *p* ≤ 0.001	*F*_(1,2380)_ = 322.112, *p* ≤ 0.001
	*η2* = 0.037	*η2* = 0.027	*η2* = 0.119
People with diabetes (*N* = 206)	5.80 (5.30)	4.03 (4.19)	75.53 (18.97)
People without diabetes (*N* = 2180)	2.97 (3.67)	2.35 (2.87)	92.87 (11.21)
BMI class	*F*_(2,2380)_ = 20.537, *p* ≤ 0.001	*F*_(2,2380)_ = 23.007, *p* ≤ 0.001	*F*_(2,2380)_ = 28.593, *p* ≤ 0.001
	*η2* = 0.017	*η2* = 0.019	*η2* = 0.023
Normal weight (*N* = 1203)	2.98 (3.62)	2.37 (2.84)	93.50 (10.70)
Overweight (*N* = 986)	3.12 (3.87)	2.39 (2.95)	90.57 (13.53)
Obesity (*N* = 197)	5.17 (5.19)	3.82 (4.14)	82.46 (18.19)
Diabetic × BMI class	*F*_(1,2380)_ = 4.018, *p* ≤ 0.05	*F*_(1,2380)_ = 8.554, *p* ≤ 0.001	*F*_(1,2380)_ = 6.727, *p* ≤ 0.01
	*η*^2^ = 0.003	*η*^2^ = .007	*η*^2^ = .006

### Anxiety—GAD-7

In regard to the GAD-7 sum score, ANOVA analyses revealed significant differences between the factor diabetes (*F*_(1, 2380)_ = 64.9, *p* ≤ 0.001, *η*^2^ = 0.027) and the factor BMI class (*F*_(2, 2380)_ = 23.0, *p* ≤ 0.001, *η*^2^ = 0.019). There was a significant interaction effect diabetes x BMI classes (*F*_(1, 2380)_ = 8.6, *p* ≤ 0.001, *η*^2^ = 0.007). Obese individuals have the strongest increase in the GAD-7 score from non-diabetes to diabetes (see Supplemental Fig. 1). Furthermore, post-hoc tests demonstrated significant higher sum score of individuals with diabetes in contrast to non-diabetic individuals in BMI class normal weight (*p* ≤ 0.001), overweight (*p* ≤ 0.05) and obesity (*p* ≤ 0.001).

### Health-related quality of life—EQ-5D-5L

As expected, the health-related quality of life (HRQOL) was significantly lower in individuals with diabetes compared to non-diabetic individuals (*F*_(1, 2380)_ = 322.1, *p* ≤ 0.001, *η*^2^ = 0.119), and ANOVA demonstrated significant differences in the factor BMI class (*F*_(2, 2380)_ = 28.6, *p* ≤ 0.001, *η*^2^ = 0.023). There was also a significant interaction diabetes x BMI class (*F*_(1, 2380)_ = 6.7, *p* ≤ 0.01, *η*^2^ = 0.006). Post-hoc tests showed significant lower scores in individuals with diabetes compared to non-diabetic individuals in all three BMI classes (normal weight (*p* ≤ 0.001), overweight (*p* ≤ 0.001) and obesity (*p* ≤ 0.001)).

### Mediating effect of BMI

**Fig. 2:**
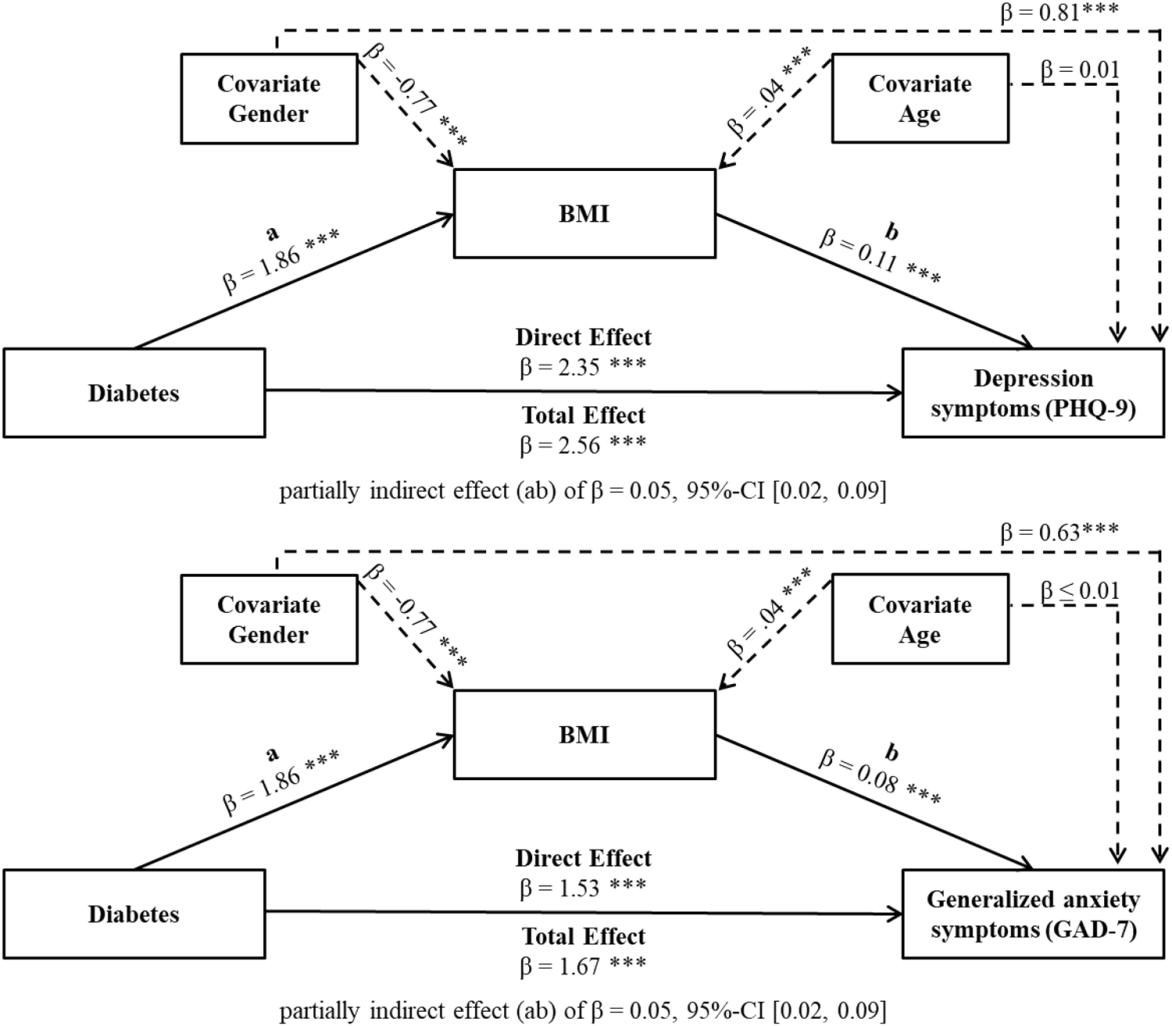
BMI as mediator of the effect of diabetes on depression symptoms and generalized anxiety symptoms including covariates gender and age. ***: *p* ≤ 0.001.

Mediation analysis demonstrated a total effect of diabetes on depressive symptoms and generalized anxiety symptoms (depression: *β* = 2.56, *p* ≤ 0.001; anxiety: *β* = 1.67, *p* ≤ 0.001). After entering the mediator into the model, diabetes predicted the mediator BMI significantly (*β* = 1.86, *p* ≤ 0.001), which in turn predicted depressive symptoms (*β* = 0.11, *p* ≤ 0.001) and generalized anxiety symptoms (*β* = 0.08, *p* ≤ 0.001) significantly. The association between diabetes and depressive symptoms as well as between diabetes and generalized anxiety symptoms is partially mediated by the BMI (depression: indirect effect (ab) β = 0.05, 95%-CI [0.02, 0.09]; anxiety: indirect effect (ab) β = 0.05, 95%-CI [0.02, 0.09]). Influence of gender (*β* =  − 0.77, *p* ≤ 0.001) and age (*β* = 0.04, *p* ≤ 0.001) on BMI must be considered. Furthermore, there was an effect of gender on parameter PHQ-9 (*β* = 0.81, *p* ≤ 0.001) and GAD-7 (*β* = 0.63, *p* ≤ 0.001). Both summarized mediation models are given in Fig. 2.

**Fig. 3:**
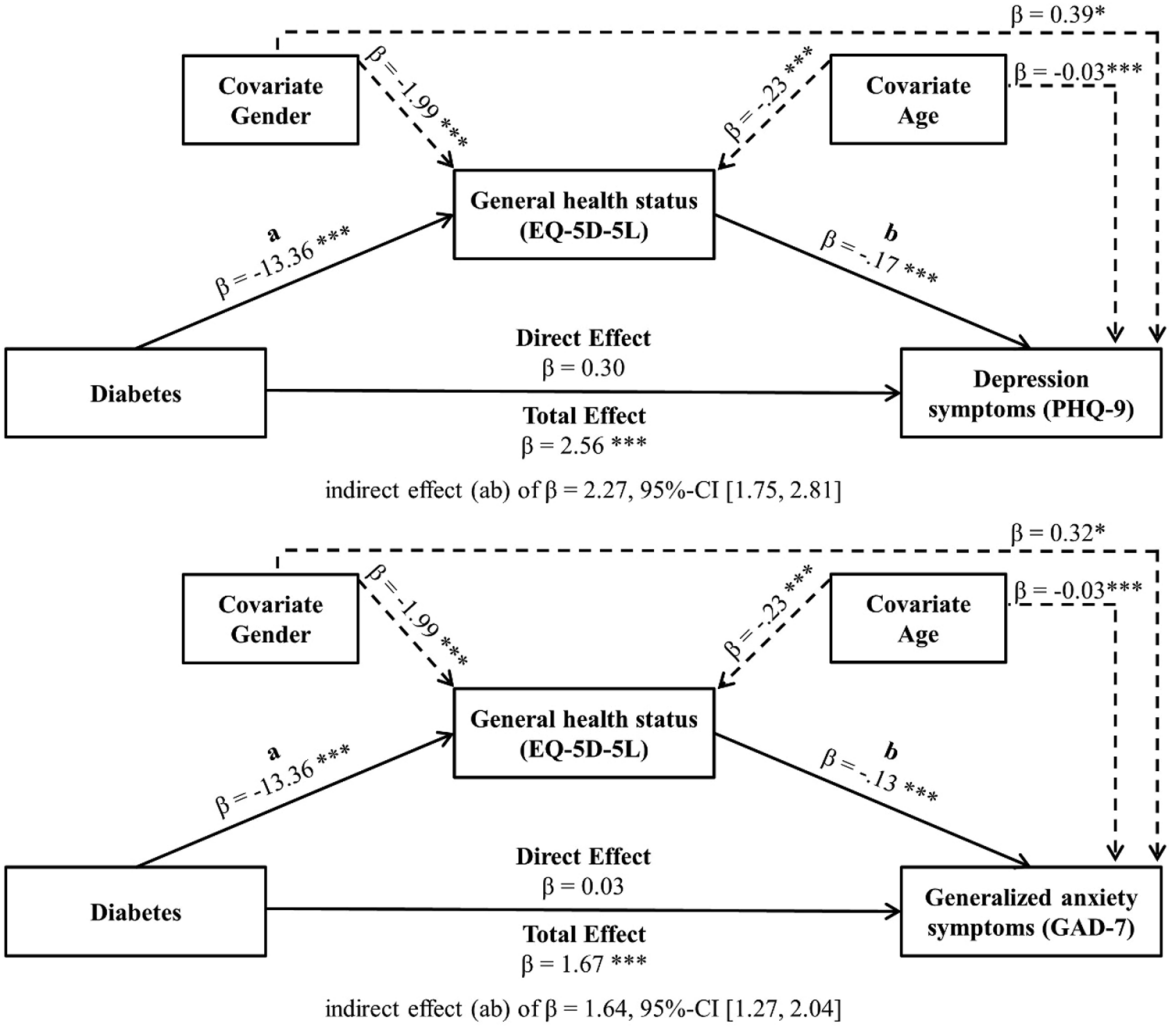
EQ-5D-5L as mediator of the effect of diabetes on depression symptoms and generalized anxiety symptoms including covariates gender and age. *: *p* ≤ 0.05; **: *p* ≤ 0.01; ***: *p* ≤ 0.001

After entering the mediator EQ-5D-5L into the model, diabetes predicted the mediator EQ-5D-5L significantly (*β* =  − 13.36, *p* ≤ 0.001), which in turn predicted depressive symptoms (β =  − 0.17, *p* ≤ 0.001) and generalized anxiety symptoms (β =  − 0.13, *p* ≤ 0.001) significantly. No direct effect for diabetes on depressive (*β* = 0.30, *p* = 0.32) or generalized anxiety symptoms was observed (*β* = 0.03, *p* = 0.90), but confidence interval of the indirect effect ab did not include zero (depression: indirect effect (ab) β = 2.27, 95%-CI [1.75, 2.81]; anxiety: indirect effect (ab) β = 1.64, 95%-CI [1.27, 2.04]). Therefore, relationship between diabetes and depressive symptoms as well as generalized anxiety symptoms is fully mediated by the EQ-5D-5L. Influence of gender (*β* =  − 1.99, *p* ≤ 0.001) and age (*β* =  − 0.23, *p* ≤ 0.001) on EQ-5D-5L must be considered. Furthermore, there was an effect of gender and age on parameter PHQ-9 and GAD-7 (see Fig. 3). Both summarized mediation models are given in Fig. 3.

## Discussion

The present study addresses the comparison of individuals with diabetes and non-diabetic individuals in depressive symptoms, generalized anxiety symptoms, and HRQOL. As expected, there were higher values in depression and anxiety as well as lower score in HRQOL in individuals with diabetes compared to non-diabetic individuals (hypothesis 1 and 2). Thereby, obese individuals with diabetes showed the highest rates in depressive symptoms (PHQ-9) and generalized anxiety (GAD-7) as well as lowest score in HRQOL (EQ-5D-5L). With regard to the mediator analyses, association between diabetes, depressive symptoms, and anxiety symptoms is partially mediated by the BMI and fully mediated by the HRQOL (hypothesis 3).

Individuals with diabetes showed higher sum scores compared to the normative data of the PHQ-9 [33] with highest difference in individuals with diabetes and obesity. It must be considered that individuals with diabetes showed PHQ-9 score between 5 and 10, which represents a mild depression [34]. PHQ-9 score ≥ 10 had a sensitivity of 88% and a specificity of 88% for major depression [34]. It must therefore be assumed that only a very small proportion in the current representative sample of the German population will suffer from depression according to DSM-IV criteria. But the present results are in accordance with previous studies [6] showing the impact of diabetes on depressive symptoms. There are different theoretical models to explain the association between diabetes and depression [35]. With regard to psychological models, there is evidence that depression is a result of the knowledge of a demanding chronic illness with burden of lifestyle changes and self-management care [36, 37]. In line with this, our data showed lower HRQOL with dimensions of mobility, self-care or usual activities in individuals with diabetes compared to non-diabetic individuals. There is evidence that poor self-rated health is a predicting factor of developing depressive symptoms in individuals with diabetes [16, 19, 20]. Concerning psychological models, it must be considered that factors of glycemic control (HbA1c), long term complications, and diabetes duration affect the physical, social and mental well-being of people [38, 39]. Future studies should include these factors regarding the link between diabetes, symptoms of depression/anxiety, HRQOL, and BMI. A further possible explanation in view of the association between diabetes and depression might be the biological mechanism [35]. Pathogenic pathways of hypothalamic-pituitary adrenal (HPA) axis dysfunction, disrupted sleep, chronic inflammation, and hippocampal dysfunction have been observed in diabetes and depression [35]. Interestingly, all of these physiological drivers are connected to obesity, which is the leading risk factor for type 2 diabetes [21]. Associations between diabetes, obesity and depression have been found[40] and there is also evidence that the link between diabetes and depression is primarily somatic-affective driven [41]. Our data provide further support of a mediating effect of the BMI and HRQOL on the association between diabetes and depressive symptoms. But it must be considered that there may be a chicken-egg problem with HRQOL and depressive symptoms, because depressive symptoms can also be considered as a mediator between diabetes and HRQOL [42, 43].

Concerning the GAD-7, higher sum scores were present in individuals with diabetes compared to normative data of representative German sample [44]. In addition, highest rates could be observed in individuals with diabetes and obesity. In line with previous studies [10, 11], the present result showed higher anxiety symptoms in diabetes. Their daily struggle with symptom-related worries such as fear of hypoglycemia, diabetic complications or increased disability could lead to development of anxiety disorders [11]. Similar to our results in depression in diabetes, lower HRQOL in individuals with diabetes compared to non-diabetic individuals could be observed. In addition, there is also evidence of physiological pathways for the development of anxiety disorders in diabetes. Physiological drivers, such as chronic inflammation [45] and HPA-axis dysregulation [46] are risk factors of anxiety, have been found in individuals with diabetes [47]. Obesity, as the leading risk factor for type 2 diabetes [21], is associated to the physiological drivers. There is also evidence of a link between diabetes, obesity and anxiety [40]. Our data showed a mediating effect of the BMI and HRQOL on the association between diabetes and anxiety symptoms.

Importantly, combination of diabetes and obesity or diabetes and low HRQOL is the worst combination with regard to the development of depressive and anxiety symptoms. Therefore, clinicians and primary healthcare providers should be aware of increased risk of increased depressive and anxiety symptoms in obese individuals with diabetes and consider routine screening among these group. Furthermore, the impact of the HRQOL within this chronic illness with burden of lifestyle changes and self-management care needs to be recognized and considered by healthcare providers. In general, there is evidence that HRQOL is a mediator in the association between BMI, depressive and anxiety symptoms [14] as well as improved HRQOL is associated to weight changes towards normal BMI [48]. Therefore, future studies should determine how interventions aimed at modifying behavioral and emotional factors will complement in current diabetes prevention strategies and diabetic treatment.

The main strength of the study is the large representative data set (N = 2386) of the German population collected by random-route sampling. In addition, the assessment of depression, anxiety, and HRQOL was conducted by standardized and reliable questionnaires. However, several limitations in the current study should be pointed out. The presence of diabetes without distinction of type 1 and type 2 was measured via self-report, but no clinical diagnose. It must be considered that individuals with type 1 diabetes are often younger and therefore have a longer history of disease, which could have a greater impact on vulnerability to anxiety and depression. Furthermore, data like glycemic control (HbA1c) and long-term complications have also an impact on symptoms of depression/anxiety and should include in future studies. Effect sizes should be included in the interpretation because small differences can be found significant in large sample size. With regard to representative data, it must be considered that diabetes affects more people in older age groups and therefore the distribution of sociodemographic variables is skewed distributed.

In conclusion, individuals with diabetes showed higher values in depressive and anxiety symptoms as well as lower HRQOL compared to non-diabetic individuals. Furthermore, risk factor overweight/obesity increased these differences in all three parameters. Future research and strategies in the public health policies among individuals with diabetes should take into account that that the association between diabetes, depression, and anxiety is mediated by BMI and HRQOL.

### Supplementary Information

Below is the link to the electronic supplementary material.

Supplementary file1 (DOCX 130 kb)
