# Supplementary material for: Depression, anxiety, and health-related quality of life in normal weight, overweight and obese individuals with diabetes: a representative study in Germany
Source: Acta Diabetol. 2024 Mar 2;61(6):725–34. doi: 10.1007/s00592-024-02248-7 (PMC11101582; doi:10.1007/s00592-024-02248-7)
Supplement: Supplementary file 1 — Supplementary file1 (DOCX 130 kb) [file 592_2024_2248_MOESM1_ESM.docx]

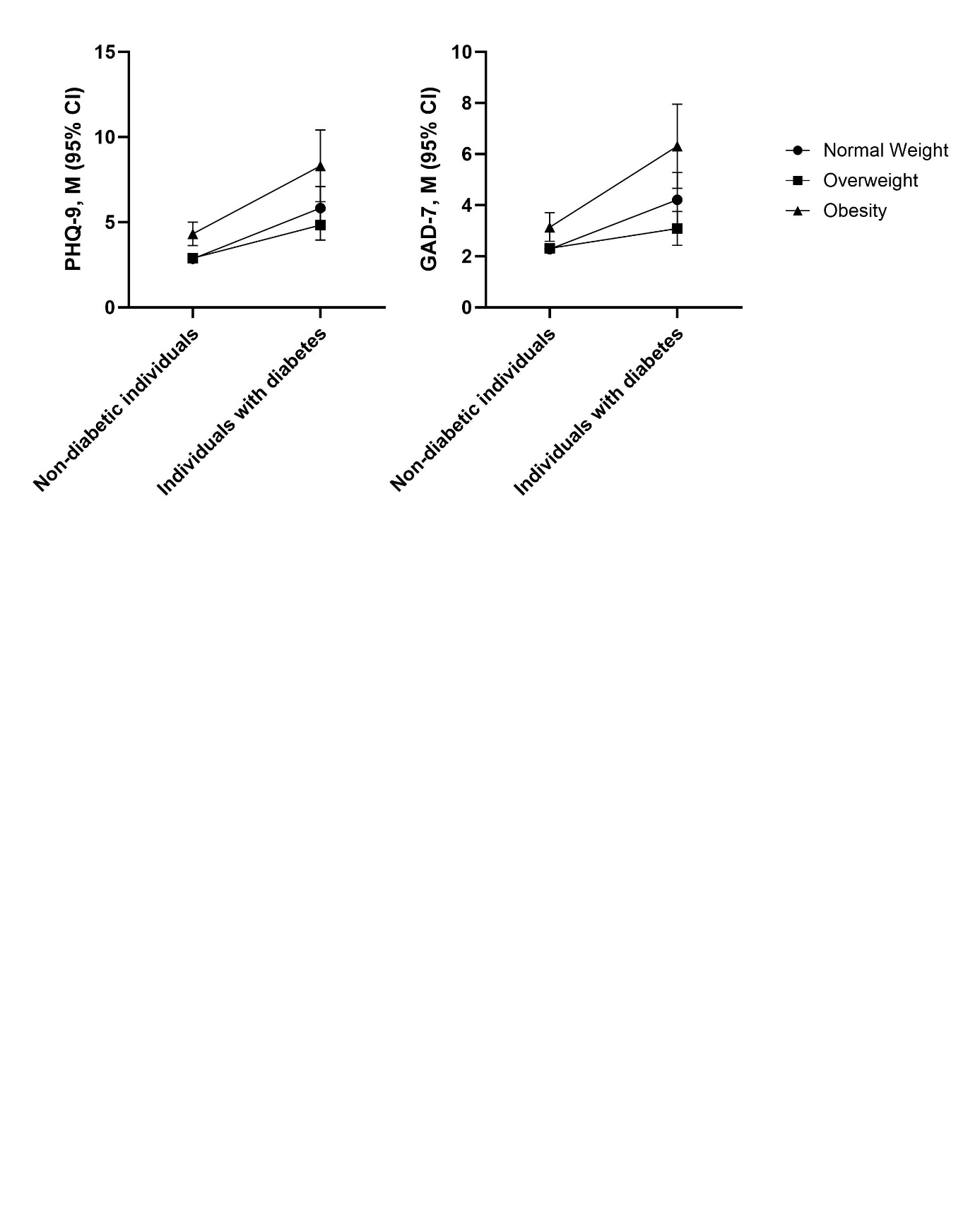


**Supplemental Figure 1.** Averaged score and 95% confidence interval in the PHQ-9 and GAD-7 of individuals with diabetes and non-diabetic individuals across different BMI classes.

M: mean; CI: confidence interval
